# Leveraging Learning Health Systems to Address Racial and Ethnic Disparities in Veteran Patient Experience

**DOI:** 10.1007/s11606-026-10205-z

**Published:** 2026-06-29

**Authors:** Evan Michael Shannon, Michelle S. Wong, Ernest Moy, Kenneth T. Jones, Jaime Boris, Donna L. Washington

**Affiliations:** 1 VA HSR Center for the Study of Health Care Innovation, Implementation and Policy, VA Greater Los Angeles Healthcare System, Los Angeles, CA USA; 2 Division of General Internal Medicine and Health Services Research, Department of Medicine, UCLA David Geffen School of Medicine, Los Angeles, CA USA; 3 Office of Health Equity, Veterans Health Administration at Time of Study, Washington, DC USA

**Keywords:** learning health system, veterans health administration, health equity, patient experience

## Abstract

Learning health systems (LHS) use continuous data-driven learning cycles to improve care delivery, but seldom apply this approach to advance health equity. This perspective describes how the Veterans Health Administration (VA) applied LHS principles through a research-operations partnership between the Office of Health Equity (OHE) and the VA Health Equity-Quality Enhancement Research Initiative (QUERI) National Partnered Evaluation Center (PEC) to address racial and ethnic disparities in patient experience among VA users. The PEC analyzed the Survey of Healthcare Experience of Patients data to identify disparities in patient experience within VA regions (Veterans Integrated Service Networks [VISNs]). To address these disparities, OHE and PEC developed a “VISN equity toolkit” that communicates VISN-specific equity gaps by population group and patient experience metrics and offers tailored equity-guided quality improvement resources for distinct patient experience domains. For instance, in one VISN, Black veterans reported lower satisfaction with office staff helpfulness and respect versus White veterans, prompting focused strategies for staff training and service recovery programs. This research-operations partnership leverages LHS principles, aligns research and healthcare priorities, maps to existing frameworks for pursuing health equity through the LHS, and tailor interventions to regional needs to improve equity in VA patient care experience.

## INTRODUCTION

The learning health system (LHS) framework bridges the gap between research and practice by embedding continuous cycles of data-driven improvement into care delivery.^1,2^ This dynamic model of iterative learning, fostering innovation and improvement at scale^1,3–6^ has the potential to advance equity within healthcare systems. Health equity^7^ is embedded within the conceptual core of LHS, with LHS core values such as inclusiveness and accessibility being inherent to equity.^8^ Additionally, the Agency for Healthcare Research and Quality (AHRQ) added “Health and Healthcare Equity and Justice” as an LHS domain in 2021.^8–13^ The PETAL (Prioritize, Engage, Target, Act, Learn) framework^8^ provides a structured approach to integrating equity into LHS and includes overarching domains (social determinants of health, sustainability, and technology) and individual components including the following: (1) *prioritizing* equity as a LHS strategic goal; (2) *engaging* communities through meaningful partnerships; (3) *targeting* inequities through a data-driven approach; (4) *acting* with data-driven and evidence-informed tailored interventions; and (5) *learning* through continuous LHS learning and improvement cycles.^8^ Table 1 provides additional details on applying PETAL to ensuring equity in LHS.

**Table 1:** Summary of PETAL (Prioritize, Engage, Target, Act, Learn) framework^8^ for Incorporating Health Equity Into Learning Health Systems, and how the VA Health Equity-Quality Enhancement Research Initiative (QUERI) National Partnered Evaluation Center’s (PEC’s) Approach to Addressing Racial and Ethnic Disparities in Patient Experience Aligned with the PETAL Framework

Components/practices	Brief description	Health Equity-QUERI PEC approach to framework alignment
Prioritize	• Ensure health equity is strategic goal of health system • Create governance for equity	• One of VA’s quintuple aim goals is equity
Engage	• Prioritize engagement of patients and their communities • Develop strategies for community engagement • Invite community members as designers and leaders, starting at the early design phase	• Veteran community members and VA administrators with experience as veterans provided input on National Veteran Health Equity Report • VA administrators with experience as Veterans conceived of dashboard visualization
Target	• Use data to characterize disparities • Use literature to develop intervention	• Analyzing patient experience data by population characteristics, then addressing racial and ethnic disparities in patient experience by VISN
Act	• Develop an action plan with community members to address contributing factors to inequities • Continuous data gathering for evaluation and monitoring	• Development of the VISN equity Toolkit, including dashboard to visualize disparities by VISN and resources to address equity gaps
Learn	• Analyze process and outcomes • Disseminate finds to key partners and in literature	• Tracking of and development of strategies to maintain dashboard engagement • Public-facing dissemination of evaluation of VISN-level differences in disparities^30﻿^

Overarching domains of PETAL include social determinants of health, sustainability, and technology

*LHS* learning health system, *VA* Veterans Health Administration, *VISN* Veterans Integrated Service Network

This perspective describes how our team of investigators and operational leaders applied LHS principles to address racial and ethnic disparities in patient experience within the Veterans Health Administration (VA). The VA is a LHS that is well-positioned to advance health equity. Our research-operations partnership between Office of Health Equity (OHE) and the VA Health Equity-Quality Enhancement Research Initiative (QUERI) National Partnered Evaluation Center (PEC) provides a valuable example of how equity can be embedded at the core of the LHS framework to address health disparities among Veterans and other populations. We first illustrate how our project aligns with the PETAL framework (summarized in Table 1), and conclude with challenges we encountered, lessons learned, and future directions in pursuit of the LHS principle of continuous quality improvement (QI).

### How the VA “Prioritizes” Health Equity

The VA serves a diverse national Veteran population, many of whom are disproportionately impacted by inequities in health outcomes and patient experience.^14–17^ In pursuit of the VA Quintuple Aim goal of Equity, the VA “Prioritizes” health equity (P of PETAL framework) through the creation of infrastructure to reduce unwarranted gaps in care and to pursue the achievement of population health. The VA has successfully used LHS foundational methods—implementation science, data science, engagement science, systems science, and policy science—to address disparities and optimize outcomes for diverse Veteran populations.^6,18^ For example, VA empowers researchers to partner with clinicians and administrators to co-design interventions, reflecting the LHS emphasis on collaborations between clinical operations partners and researchers or systems science.^6,18,19^ Further, QUERI and VA’s Health Systems Research program provide dedicated funding opportunities to foster partnerships between researchers, clinical operations, and program office leaders to amplify health equity work across VA Medical Centers.

The VA Health Equity-QUERI PEC is an example of a researcher-clinical operations-program office leader partnership developed to improve health outcomes, quality, and patient experience across the VA. The Office of Health Equity was created by the VA in 2012 to coordinate and champion national efforts to support equitable health for all Veterans. To provide data on healthcare inequities by race and ethnicity, among other sociodemographic variables, the VA Health Equity-QUERI PEC produced the National Veteran Health Equity Report (NVHER), first in 2016 and then updated in 2021.^20^ The NVHER provides extensive data on health equity inequities by a variety of sociodemographic variables and has been widely used by clinical operations partners to guide policy and practice decisions.

### “Engaging” the VA Community to Advance Equity

During the development of the NVHER and related chartbooks, input from diverse Veteran community members and VA minority Veteran coordinators (many of whom are Veterans) was actively sought during presentations to key partners during the National Minority Veterans Summit and Center for Minority Veteran meetings. We were especially interested in hearing from Veterans if the findings resonated with their personal experiences as VA users and determining if the data visualization was clearly interpretable by Veterans. Input from Veteran VA leaders was obtained to provide context for findings and to triangulate findings with those from other sources of Veteran perspectives. Feedback from these varied Veteran community representatives was incorporated into final versions of the report. Later, in the “Action” phase and development of the patient experience disparity dashboard, we used a visualization format based on direct feedback from VISN-level leaders, many of whom were also Veterans.

Our ability to fully engage Veteran community members as collaborators and co-leaders in this work was constrained due to the acute phase of the COVID-19 pandemic, which limited capabilities for in-person meetings and diffused resources from the PEC to ensure preservation of an equitable response to the pandemic by the VA. We also did not earmark funds in the PEC’s budget to be used to support Veteran community member involvement in the analytic phase of the project.

### “Targeting” Racial and Ethnic Disparities in Patient Experience by Geographic Region

Given prior research that identified worse patient experiences among Black and Hispanic compared to White Veterans across multiple care settings,^20–26^ the NVHER provided in-depth data on these differences within the VA. The NVHER used data from VA’s Survey of Healthcare Experiences of Patients (SHEP)-Patient-Centered Medical Home (PCMH),^20^ which is based on the industry-standard Consumer Assessment of Health Provider Survey (CAHPS),^27^ to characterize these differences. The NVHER identified racial and ethnic disparities across multiple measures of patient experience for minoritized groups compared to white racial and ethnic groups.

Given the likelihood of variation in racial and ethnic disparities due to local geographic contexts,^28,29^ we expanded upon the NVHER findings by characterizing regional differences in disparities.^30^ The VA is organized into 18 regions across the USA named Veterans Integrated Service Networks (VISNs). VISNs are regional systems of VA healthcare sites that meet the local needs of Veterans in their catchment area. Exploration of VISN variation allows VISN leaders to identify disparities within their VISN and prioritize resources and efforts to close these gaps.

We relied on the LHS foundational method of data science and LHS core principle of scientific integrity to apply scientific rigor and ensure the validity of our findings. We examined data from the VA SHEP-PCMH survey from fiscal years 2016–2019. We applied dual criteria to define meaningful differences between racial and ethnic minoritized and White groups within each VISN. These criteria were a statistically significant difference in positive patient experience, controlling for patient age and sex, at a two-tailed *p*-value < 0.05, with a relative difference value of at least ± 10%. This approach is consistent with the AHRQ National Healthcare Quality Disparities Report.^31^ Relative difference is a preferable measure of disparity/advantage over absolute difference when the criterion for what is meaningful and important varies depending on where the historically advantaged group is on the outcome or experience measure. We omitted other covariates known to be associated with race and ethnicity (e.g., socioeconomic status) so that our findings were representative of different racial and ethnic groups.^31^ If the minoritized group experienced meaningfully worse care, this was considered a disparity; meaningfully better care was considered an advantage; and no meaningful difference was considered equivalent. To address issues of smaller sample sizes at the VISN level for several race and ethnicity groups and the risk for type II error, we reported equivalence of patient experiences only for comparisons with ≥ 80% power to detect difference.

### “Acting” on Data Through Toolkit Development

**Figure 1:**
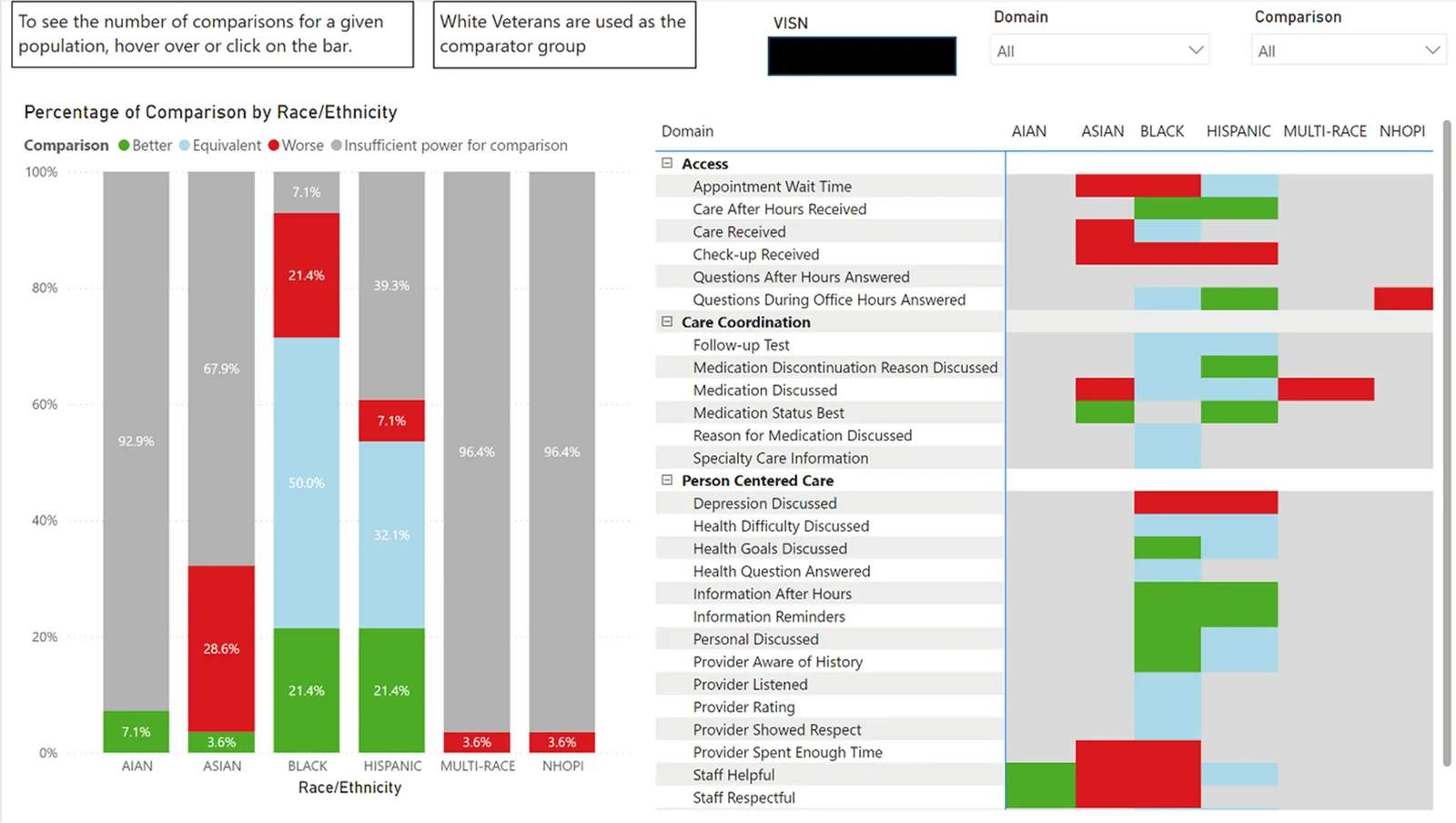
Example of a view of a VISN-level summary report from the patient experience equity dashboard. The panel on the left displays a summary of the comparisons for each racial and ethnic minoritized group compared with White Veterans; the panel on the right shows comparisons for each measure. Abbreviations: AIAN, American Indian, Alaska Native; NHOPI, Native Hawaiian, Other Pacific Islander; VISN, Veterans Integrated Service Network.

In partnership with the OHE, we leveraged health information technology and informatics (PETAL overarching domain of “technology”) to develop a "VISN equity toolkit," which includes a patient experience equity dashboard and resource summaries to address patient experience disparities. The dashboard visualizations display each age-and-sex-adjusted SHEP measure as better, equivalent, or worse for each minoritized race-ethnicity group compared with White Veterans for the compiled 2016–2019 SHEP data. Options for visualizing equity gaps include VISN-level grids displaying all 28 measures by racial-ethnic group, with color coding to illustrate the results of comparisons (Fig. 1), and cross-VISN comparison grids that display comparison results by VISN for each selected racial-ethnic group (not shown). The tool is accessible on a VA intranet SharePoint site for use by VISN and healthcare system administrators. This dashboard is similar to tools developed in other LHS health equity initiatives,^32^ such as VA’s Primary Care Equity Dashboard, which displays quality metrics by sociodemographic group.^33^

**Figure 2:**
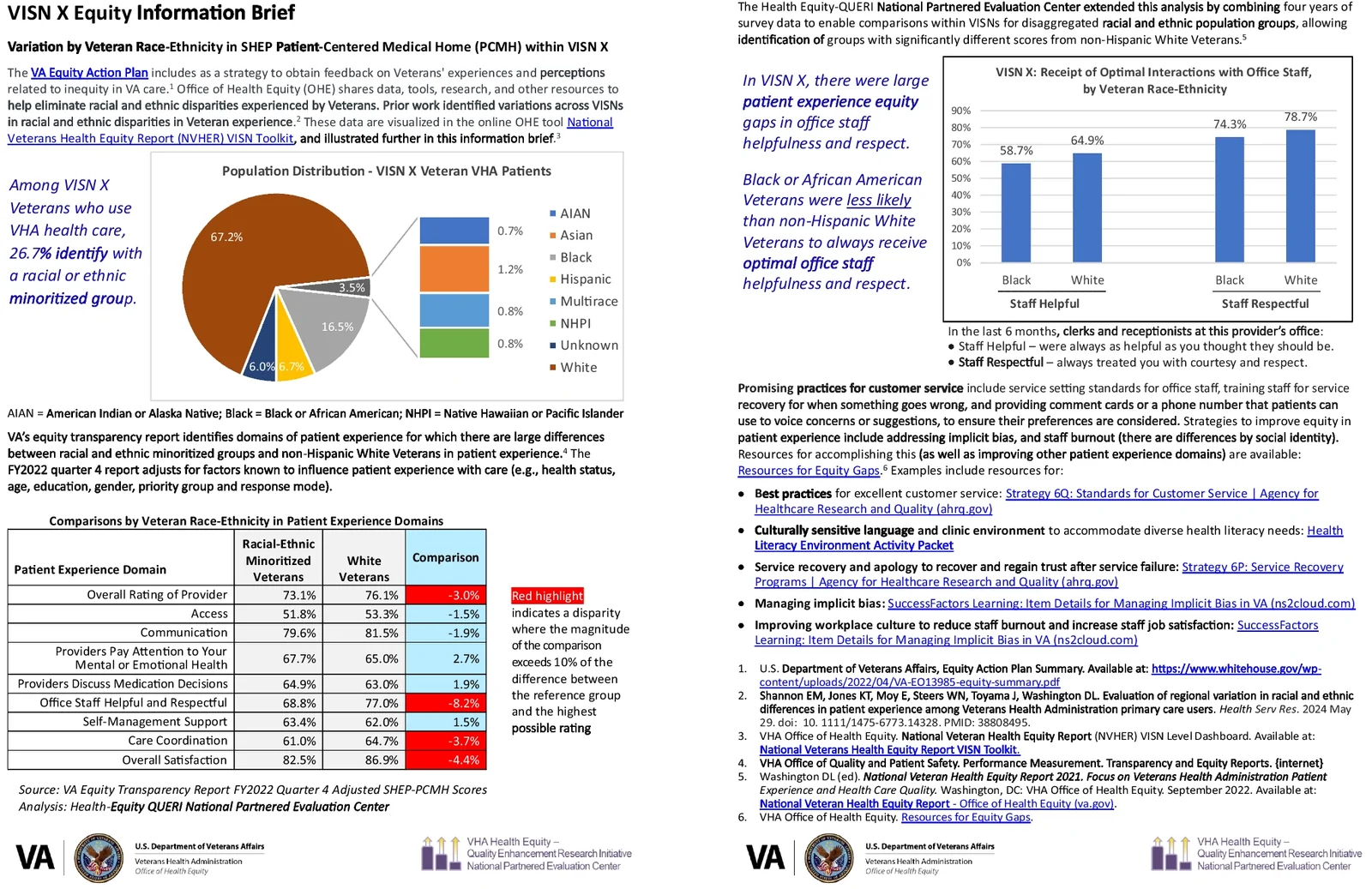
Example VISN Equity Information Brief summary and resource guide (deidentified). Abbreviations: AIAN, American Indian, Alaska Native; NHPI, Native Hawaiian, Pacific Islander; QUERI, Quality Enhancement Research Initiative; SHEP, Survey of Healthcare Experiences of Patients; VISN, Veterans Integrated Service Network.

In response to the equity gaps identified, the VISN equity toolkit links the dashboard with compiled resources to address equity gaps. We drew from the VA’s SHEP toolkits, which include recommendations and resources for practices and tools drawn from existing VA projects and interventions, Diffusion of Excellence and VA Innovation Network recommendations, patient advocacy groups, and academic journals. For patient domains (i.e., access, person-centered care, and care coordination) with an identified disparity, we reviewed SHEP toolkit resources to identify those that could be tailored to the group experiencing the equity gap. For instance, in VISNs where Black veterans reported lower satisfaction with office staff helpfulness and respect versus White veterans, focused strategies include staff training and service recovery programs.^34,35^ Next, in collaboration with OHE, we reviewed all OHE-funded equity-guided improvement projects to identify interventions that improve racial and ethnic minoritized patients’ healthcare experiences (e.g., shared decision-making tools). As a toolkit component, we developed summaries of our analysis findings and available resources in “Equity Information Briefs” for VISN administrators (Fig. 2).

### “Learning” and Improving

Moving forward, there are several actions to improve the toolkit and ensure that it meaningfully addresses disparities in VA patient experience. The first key step is OHE’s current toolkit dissemination to VISN administrations and VA sites, ensuring its adoption and practical application. Since the VISN dashboard has been rolled out, we have noted modest usage, particularly after presentations on the dashboard at leadership meetings, and are devising methods for maintaining engagement and tracking toolkit use, such as incorporating it into OHE-sponsored QI trainings and learning communities. Engagement with Veterans to refine the toolkit resources based on their feedback will involve the LHS engagement science foundational method and align with the frameworks’ emphasis on patient and community engagement. To obtain feedback on the dashboard findings and toolkit resources directly from Veterans, we plan to engage our local Health Systems Research Center of Innovation Veteran Engagement Team (VET), a diverse team of local Veterans that imparts Veteran-based knowledge and perspectives on QI efforts, as consultants.^36^ Our VET has an established record of working closely with local VA researchers, often through continual engagement across multiple meetings throughout project stages, and its members are compensated for their time. If resources allow, we will also consult with other Veteran Engagement Groups across the country to ensure the feedback we receive incorporates nationally representative Veteran voices. This will help ensure recommended strategies align with the lived experiences of Veterans from diverse backgrounds.

Limitations of our work include our use of data that was collected over five years ago (update planned) and the absence of benchmark values for patient experience; the addition of benchmarks would complement the dashboard by monitoring receipt of high-quality patient experiences by all Veteran groups. Implementation science methods in concert with community engagement could also be used to develop novel interventions to reduce disparities in patient experience. As our LHS continues to evolve, we strive to continue to “practice a growth mindset”^11^ to create an environment that allows for questioning assumptions about the status quo and fosters curiosity, innovation, and creativity in the shared pursuit of equity for our most underserved Veterans.

Through our process, we are learning how integrating health equity into the LHS framework presents complex challenges. One inherent challenge lies in using broad racial and ethnic categories to define groups, which risks oversimplifying identities and ignoring the intersectionality of individual experiences. This challenge relates to the PETAL “Target” health disparities principle, which emphasizes evidence-based targeting of disparities while being sensitive to nuanced social constructs. Similarly, we had to balance statistical regression methods that control for all potential confounders to isolate the effect of “race” (i.e., racism) in our analysis, with limiting covariate adjustment, with the goal of understanding the lived experiences of racial and ethnic groups. Additional challenges include limited representation of smaller racial and ethnic groups, which affected decision-making about the thresholds needed to generate actionable insights at the VISN level. These issues highlight the need for robust data systems that capture and analyze intersectional identities.

While the VA is unique among healthcare systems in that many high-ranking officials have lived experience as Veterans, and Veteran community members and Veteran VA administrators were engaged throughout the NVHER and dashboard development, a missed opportunity lies in the depth of community engagement to date and in our plans to consult with Veterans Engagement Groups. This shortcoming connects to the “Engage” principle of PETAL, which stresses the importance of involving affected communities in decision-making and analysis. Ideally, embedding health equity into LHS would involve community members leading or co-leading the work^11^ and co-developing research questions and aims with researchers from inception to conclusion and sustainment. This could involve linking dashboard findings with community-identified patient experience metrics and resources, as we identified resources within VA’s network of researchers and resources. Achieving this depth of engagement necessitates time to forge strong and meaningful relationships with community members and dedicated funding to compensate community members for their involvement in health equity projects. Further, we could also have emphasized engaging beyond the healthcare system in the toolkit resources,^11^ to consider how local, state, and other geographic and community contexts external to VA could contribute to narrowing patient experience disparities.

## CONCLUSIONS

This work highlights the transformative potential of embedding health equity into the core of VA’s LHS. Our research-operations partnership is a case example that aligns with the PETAL framework to address equity gaps. The development and application of the VISN equity toolkit underscore the importance of combining rigorous data analytics with actionable strategies that address systemic disparities at regional and national levels. Moving forward, the VA’s ongoing refinement and implementation of these tools can serve as a replicable model for other healthcare systems striving to align their operational priorities with health equity goals.
